# Warming, shading and a moth outbreak reduce tundra carbon sink strength dramatically by changing plant cover and soil microbial activity

**DOI:** 10.1038/s41598-017-16007-y

**Published:** 2017-11-22

**Authors:** Mathilde Borg Dahl, Anders Priemé, Asker Brejnrod, Peter Brusvang, Magnus Lund, Josephine Nymand, Magnus Kramshøj, Helge Ro-Poulsen, Merian Skouw Haugwitz

**Affiliations:** 1 Center for Permafrost (CENPERM), Department of Geosciences and Natural Resource Management, University of Copenhagen, Øster Voldgade 10, DK-1350 Copenhagen K, Denmark; 2 Department of Biology, University of Copenhagen, Universitetsparken 15, DK-2100 Copenhagen Ø, Denmark; 3 Arctic Research Centre, Department of Bioscience, Aarhus University, Frederiksborgvej 399, DK-4000 Roskilde, Denmark; 4 Department of Environment and Mineral Resources, Greenland Institute of Natural Resources, Box 570, DK-3900 Nuuk, Greenland

## Abstract

Future increases in temperature and cloud cover will alter plant growth and decomposition of the large carbon pools stored in Arctic soils. A better understanding of interactions between above- and belowground processes and communities of plants and microorganisms is essential for predicting Arctic ecosystem responses to climate change. We measured ecosystem CO_2_ fluxes during the growing season for seven years in a dwarf-shrub tundra in West Greenland manipulated with warming and shading and experiencing a natural larvae outbreak. Vegetation composition, soil fungal community composition, microbial activity, and nutrient availability were analyzed after six years of treatment. Warming and shading altered the plant community, reduced plant CO_2_ uptake, and changed fungal community composition. Ecosystem carbon accumulation decreased during the growing season by 61% in shaded plots and 51% in warmed plots. Also, plant recovery was reduced in both manipulations following the larvae outbreak during the fifth treatment year. The reduced plant recovery in manipulated plots following the larvae outbreak suggests that climate change may increase tundra ecosystem sensitivity to disturbances. Also, plant community changes mediated via reduced light and reduced water availability due to increased temperature can strongly lower the carbon sink strength of tundra ecosystems.

## Introduction

Increasing temperatures, increasing cloud cover and changing soil water availability are part of the predictions for the future climate in the Arctic^1,2^. During the last decades, this region has experienced some of the highest rates of warming, which have altered plant communities^3,4^ and changed soil carbon (C) dynamics^5,6^. Since Arctic soils are estimated to contain 50% of the global soil organic C pool^7,8^, understanding the effect of climate changes on the mobilization of these C stocks is of great importance for predicting whether Arctic ecosystems will serve as C sinks or sources in the future.

Climate models predict continuous high warming rates in the Arctic and therefore longer growing seasons^2^. Based on satellite observations of tundra ecosystems, warming increases net primary production^9^. Furthermore, warming enhances the abundance of shrubs in the Arctic at the expense of grasses and mosses, primarily by increasing the abundance of deciduous shrubs^4,10^, although warming is also expanding the distribution of evergreen ericaceous shrubs^11^. In contrast to mosses and Arctic grasses, deciduous and ericaceous shrubs form mycorrhizal symbioses with soil fungi to facilitate their nutrient uptake^12^. Since warming is expected to increase plant growth and thus enhance the demand of plants for nutrients^13^, warming may favor an increased mycorrhizal colonization of shrub roots^14,15^. Warming can also affect Arctic soil microbes directly by altering the microbial community composition^16,17^, shifting the fungal to bacterial ratio^18^, and increasing microbial activity^17^. This may result in a faster turnover of organic matter and a higher soil nutrient availability^19,20^, however, possibly increasing C losses from Arctic soils, with associated feedback effects on the climate system.

Increased shrub density and height in tundra ecosystems not only reduces species richness of the plant community below the shrubs^21^. A denser canopy also transmits less solar radiation to the soil, thereby possibly reducing soil temperature^22,23^. Combined with a reduced incident radiation due to increased cloud cover^2^, this may further offset soil warming. A meta-analysis based on long-term ecosystem-scale experiments in the Arctic did not reveal any effects of shading on aboveground plant biomass^24^. However, reduced plant productivity after nine years of shading^25^ together with a tendency towards lower colonization of fine roots by ericoid mycorrhizal fungi^26^, a lower fungal abundance and an altered soil bacterial community after 15 years^18^, suggest a slow, yet significant, shading response in Arctic tundra ecosystems.

Mature ecosystems are considered to be less sensitive to climate change than disturbed, early-succession ecosystems^27^, where the vegetation are more sensitive to environmental changes^28,29^. Following a severe insect outbreak, the vegetation is in a state of short-term succession during which the ecosystem may be less resistant to the effects of climate change. In the Arctic, insect outbreaks can lead to defoliation even at regional scales^30,31^ and are known to change C cycling in subarctic forests by changing soil fungal communities^32,33^. The noctuid moth *Eurois occulta* is widely distributed across the Arctic. In Greenland, *E*. *occulta* outbreaks have been documented in peat cores as far back as the 15^th^ century^34^, and several outbreaks have been recorded in Southwestern Greenland within the last century^35^.

Plant communities are responsible for the amount and quality of organic C entering the soil (litter and root exudates), whereas soil microorganisms control the rates of mineralization and participate in both competitive and symbiotic interactions with plants^36–38^. The strong coupling between plant and soil microbial communities makes studies that include both above and belowground processes essential for understanding ecosystem feedbacks to climate change. Future climate change could potentially decouple these interactions through differences in response rates between microorganisms and plants^39^, which are likely associated with differences in generation time between plants and microorganisms^39,40^. Still, to our knowledge no other studies in the Arctic have included detailed analyses of both above- and belowground processes, the joint responses of plant and soil microbial community compositions to climate change, and the effects of a severe natural disturbance like an insect outbreak.

The main purpose of this study was to combine analyses of above- and belowground processes and their interdependence in a tundra ecosystem where warming and shading were manipulated to mimic future climate. This was done in an *Empetrum*- and *Salix*-dominated Arctic dwarf-shrub heath in West Greenland by continuous measurements of ecosystem CO_2_ fluxes each year during seven growing seasons combined with analyses of the fungal community, microbial activity, soil nutrient availability, and vegetation composition after six years of treatment. We hypothesized that warming enhances the C flow through the ecosystem due to higher photosynthetic activity, altered fungal community composition and enhanced soil microbial activity, the latter of which will increase nutrient availability and the overall C sequestration at the heath site^41,42^. In contrast, we expected shading to decrease the C flow at the experimental site due to a reduced photosynthetic activity, a reduction in vascular plant cover, lower soil microbial activity and an altered fungal community composition. A large larvae outbreak of *Eurois occulta* in the fifth treatment year set back the vegetation by inhibiting the production of leaves, buds and flowers^43^. The experiment was accordingly also used to assess the resilience of the manipulated plots to this natural disturbance. Arctic dwarf-shrub heaths are widespread across the Arctic. We therefore expect the results from this study to significantly increase our understanding of the sensitivity of above- and belowground interactions in terrestrial Arctic ecosystems to climate change and natural disturbances, and the implications for the CO_2_ flux in these ecosystems.

## Results

### Soil moisture and temperature

**Figure 1:**
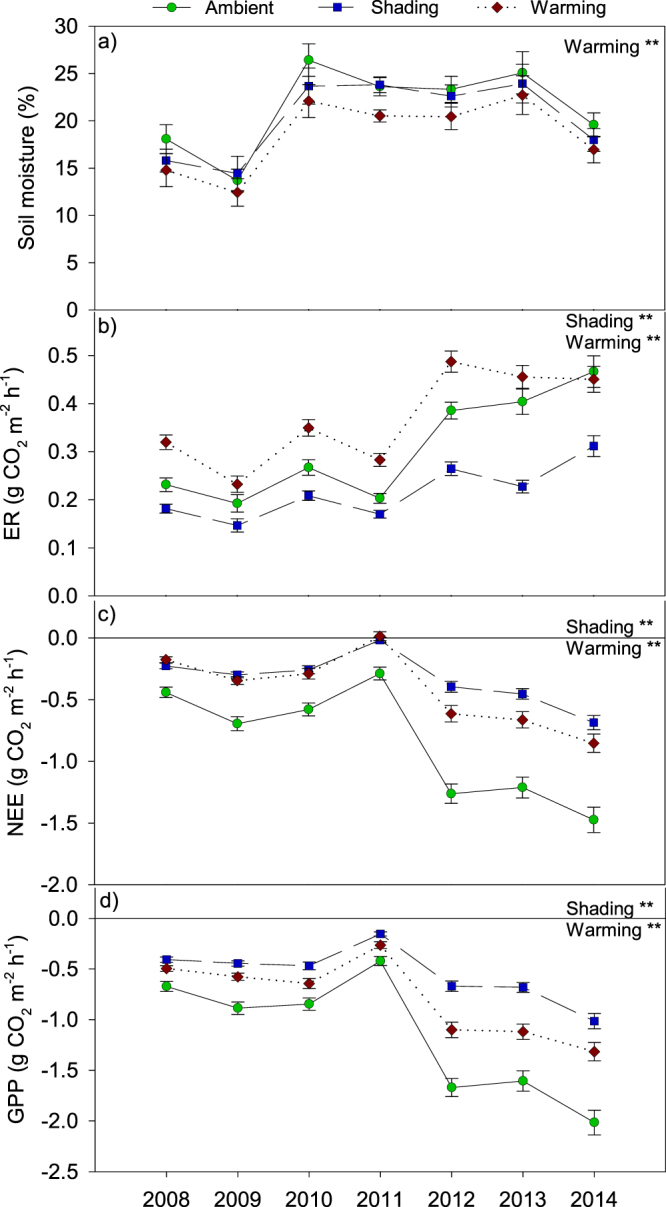
Average soil moisture (**a**), ecosystem respiration (ER) (**b**), net ecosystem exchange (NEE) (**c**), and gross primary production (GPP) (**d**) under mid-day conditions during the growing season from 2008–2014. An outbreak of the noctuid moth *Eurois occulta* affected the experimental site in 2011. Data are mean values obtained from six replicates ± s.e.m. The treatments were ambient, shading and warming. The statistical significant effects of the treatments are indicated: **p < 0.01.

Soil moisture content (% of dry weight soil) during the growing season from 2008 to 2014 fluctuated between 13% and 27% with 2008, 2009, and 2014 being the driest years (Fig. 1a). The soil moisture content was consistently lower in warming plots (p < 0.01) compared to shading and ambient plots (Fig. 1a). In addition, the days with soil moisture content below 10% of dry weight soil were markedly higher in warming plots (276 days) compared with ambient and shading plots (176 and 190 days, respectively, Supplementary Fig. S1). Neither warming nor shading affected the average soil temperature across the year or sampling period in 2013 (Supplementary Fig. S2a,b). However, for July 2013, warming increased soil temperature by 0.5 ± 0.03 °C during the day (p < 0.05), whereas shading decreased soil temperature by 0.6 ± 0.03 °C from mid-day throughout the night (p < 0.01; Supplementary Fig. S2c). This pattern showing an increase in soil temperature in the warmed plots and a decrease in temperature in shaded plots during summer was manifest in the first year of the experiment and was consistent in other years with a full set of soil temperature data throughout the year (2008 and 2011).

### Outbreak of *Eurois occulta*

Larvae of *E*. *occulta* were observed mainly in 2010 and 2011. During 2010, 31 larvae were caught in the pitfall traps, but in summer 2011 numbers increased dramatically peaking in July with 1842 trapped larvae. The outbreak was short-lived, as only 56 larvae were caught in August 2011 and none were caught in 2012 and 2013, The outbreak resulted in a near-complete defoliation of the plots and plant reproduction was very low, e.g. no *Salix glauca* catkins were observed in 2011.

### CO_2_ fluxes and vegetation cover

An average based on the CO_2_-flux measurements from 2008 to 2014 showed that all plots in the dwarf-shrub heath served as a sink for atmospheric CO_2_ from the end of June to the end of September at the time of the measurements (midday). However, in 2011 the average net ecosystem exchange (NEE) was close to zero in both warming and shading plots and some warming plots showed overall positive NEE values indicating a CO_2_ source (Fig. 1c). After NEE was set back by the *Eurois occulta* outbreak in 2011 (Supplementary Fig. S3), NEE became more negative (i.e. increased C uptake) in following years in all plots (Fig. 1c). The climate change treatments also affected NEE. Thus, the shading (p < 0.01) and warming (p < 0.01) plots had significantly reduced net C uptake relative to ambient plots (Fig. 1c), which was concurrent with a lower gross primary production (GPP) in both treatments (p < 0.01) (Fig. 1d). Ecosystem respiration (ER) from 2008 to 2014 increased throughout the period. Shading decreased ER (p < 0.01), whereas warming increased ER (p < 0.01), except in 2014 when ambient and warming plots did not significantly differ (Fig. 1b). Taken together, the average midday CO_2_ accumulation was reduced by 61% in shading plots (p < 0.01) and by 51% in warming plots (p < 0.01) during the main growing season from 2008 to 2014.

**Figure 2:**
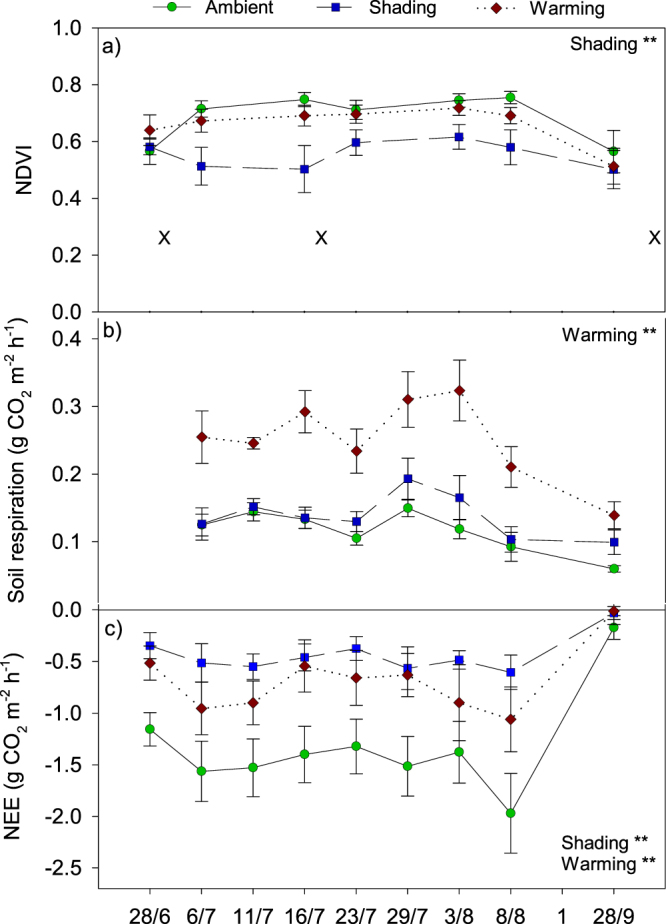
Normalized difference vegetation index (NDVI) (**a**), soil respiration (**b**), and net ecosystem exchange (NEE) (**c**) measured under mid-day conditions across the growing season 2013. The “X” in plot (**a**) marks the soil sampling dates July 1, July 19 and September 29. Data are mean values obtained from six replicates ± s.e.m. The treatments were ambient, shading and warming. The statistical significant effects of the treatments and sampling day are indicated: **p < 0.01.

After six years of manipulations, shading resulted in a significantly lower NDVI (p < 0.01; Fig. 2a). This correlated with the estimated aboveground plant biomass (p < 0.05, r^2^ = 0.49, Supplementary Fig. S4), which was also lower in shaded plots (p < 0.01; Table 1). The coverage of the dominant plant species *E*. *hermaphroditum* decreased by 38% (p < 0.05; Table 2). Additionally, warming plots had a reduced coverage of *E*. *hermaphroditum* by 36% (p < 0.05; Table 2). In both treatments, the cover of *Salix glauca*, mosses and organic crust increased, but were not significantly different from the ambient plots. As for the annual averages, NEE was significantly higher at all sampling days throughout the 2013 season in both warming (p < 0.01) and shading plots (p < 0.01), which meant a lower CO_2_ uptake in these plots compared to ambient plots (Fig. 2c).

**Table 1:** Soil properties and aboveground plant biomass after six years of climate change manipulations. Data are mean values obtained from six replicates ± s.e.m. The treatments were; *A* ambient, *S* shading, and *W* warming. The statistical significant effects of the treatments and sampling day are indicated: **p < 0.01; ns = non-significant. Arrows indicate the direction of the change.

	*A*	*S*	*W*	*Statistical significance*
*Soil pH*	4.8 ± 0.1	4.7 ± 0.1	4.8 ± 0.1	ns
*Total soil C (mg C g* ^−1^ *soil*)	172.6 ± 28.3	231.6 ± 39.8	262.8 ± 58.8	ns
*Total soil N (mg N g* ^−1^ *soil)*	7.7 ± 0.9	9.9 ± 1.3	9.8 ± 1.8	ns
*Total soil P (mg P g* ^−1^ *soil)*	0.7 ± 0.1	0.9 ± 0.1	0.8 ± 0.1	ns
*Soil C:N ratio*	21.6 ± 1.6	23.3 ± 1.9	27.0 ± 1.4	↑ W**
*Aboveground plant biomass (g dry weight m* ^−2^ *)*	625.7 ± 32.4	459.0 ± 52.3	555.7 ± 40.9	↓ S**

**Table 2:** Percentage cover of plant species, mosses, lichens, and organic crust, following six years of climate change manipulations estimated by pin-point analysis (n = 6 ± SE). The treatments were; A ambient, S shading, and W warming. The statistical significant and near-significant effects of the treatments are indicated: *p < 0.05; ns = non-significant. Arrows indicate the direction of the change. In addition, the type of fungal symbiont hosted by the plant species is indicated.

	*Fungal symbiont*	*A*	*S*	*W*	*Statistical significance*
*Carex bigelowii*	—	5.0 ± 2.6	2.8 ± 2.3	1.6 ± 1.2	ns
*Lycopodium annotinum*	—	1.4 ± 0.8	3.2 ± 2.0	0.8 ± 0.6	ns
*Lycopodium alpinum*	—	0.0 ± 0.0	1.4 ± 1.2	0.0 ± 0.0	ns
*Poa sp*.	—	0.8 ± 0.6	4.0 ± 2.5	0.7 ± 0.7	ns
*Empetrum hermaphroditum*	Ericoid mycorrhizal	52.2 ± 4.3	32.4 ± 9.4	33.3 ± 4.1	↓ S*, ↓ W*
*Ledum groenlandicum*	Ericoid mycorrhizal	0.0 ± 0.0	0.3 ± 0.3	0.0 ± 0.0	ns
*Vaccinium uliginosum*	Ericoid mycorrhizal	4.2 ± 2.3	1.6 ± 0.5	4.7 ± 3.1	ns
*Betula nana*	Ectomycorrhizal	3.0 ± 1.8	0.2 ± 0.2	2.4 ± 2.4	ns
*Polygonum viviparum*	Ectomycorrhizal	0.0 ± 0.0	0.0 ± 0.0	0.1 ± 0.1	ns
*Salix glauca*	Ectomycorrhizal	8.2 ± 1.2	8.7 ± 2.3	13.4 ± 2.8	ns
*Lichens*	—	4.7 ± 2.0	5.1 ± 3.0	5.9 ± 1.9	ns
*Mosses*	—	9.5 ± 3.0	15.8 ± 4.0	16.3 ± 3.0	ns
*Organic crust*	—	11.0 ± 1.7	24.2 ± 4.1	20.8 ± 5.3	ns
*Bed rock*	—	0.1 ± 0.1	0.3 ± 0.3	0.0 ± 0.0	ns

### Soil pH, nutrient pools, microbial abundance, and microbial activity

After six years of manipulations, the soil pH averaged to 4.8 ± 0.4 and did not vary among treatments (Table 1). In addition there was no treatment effect on total soil C, N, and P, although total soil C:N ratio increased due to warming (p < 0.01; Table 1).

Soil nutrient concentrations were affected by sampling time with NO_3_^−^ (p < 0.01) and PO_4_^3−^ (p < 0.01) peaking in mid-season on July 19^th^, whereas the highest concentrations of dissolved organic carbon (DOC) (p < 0.01) and dissolved organic nitrogen (DON) (p < 0.05) were found in the end-season on September 29^th^ (Table 3). Furthermore, shading increased the concentrations of NH_4_^+^ (p < 0.01), PO_4_^3−^ (p < 0.01), and DON (p < 0.05), while warming increased the concentrations of NO_3_^−^ (p < 0.05), PO_4_^3−^ (p < 0.05), and DON (p < 0.05) across season (Table 3).

**Table 3:** Soil nutrient concentrations, fungal and bacterial abundance after six years of climate change manipulations. Data are mean values obtained from six replicates ± s.e.m. Soil samples were collected on July 1, July 19, and September 29 2013. The treatments were ambient (A), shading (S), and warming (W). The statistical significant effects of the treatments and sampling day are indicated: *p < 0.05; **p < 0.01. Arrows indicate the direction of the change.

	July 1			July 19			September 29			Statistical significance
	Ambient	Shading	Warming	Ambient	Shading	Warming	Ambient	Shading	Warming	
NO_3_^−^ (µg g^−1^ soil)	0.01 ± 0.01	0.01 ± 0.01	0.02 ± 0.01	0.22 ± 0.04	0.30 ± 0.06	0.37 ± 0.07	0.04 ± 0.01	0.06 ± 0.01	0.05 ± 0.01	Day** ↑ W*
NH_4_^+^ (µg g^−1^ soil)	0.33 ± 0.03	1.13 ± 0.34	0.34 ± 0.14	0.14 ± 0.02	0.95 ± 0.35	0.33 ± 0.09	0.41 ± 0.17	1.65 ± 0.58	1.03 ± 0.39	↑ S**
PO_4_^3−^ (µg g^−1^ soil)	0.55 ± 0.05	0.89 ± 0.15	0.88 ± 0.17	1.01 ± 0.18	1.38 ± 0.09	1.54 ± 0.19	0.53 ± 0.17	1.18 ± 0.35	0.67 ± 0.12	Day** ↑ S**, ↑ W*
DOC^A^ (µg g^−1^ soil)	170 ± 20	213 ± 49	173 ± 37	—	—	—	257 ± 116	587 ± 74	591 ± 91	Day**
DON (µg g^−1^ soil)	7.56 ± 0.56	11.78 ± 1.82	9.54 ± 2.01	10.12 ± 1.70	12.94 ± 1.96	14.13 ± 3.17	9.77 ± 3.41	17.90 ± 4.60	17.56 ± 5.23	Day* ↑ S*, ↑ W*
Fungal abundance (10^8^ x ITS copies g^−1^ soil)	4.14 ± 1.71	2.71 ± 1.63	5.99 ± 3.49	13.48 ± 4.42	5.96 ± 2.58	4.11 ± 2.81	6.23 ± 1.21	10.02 ± 2.38	9.62 ± 3.49	Day*
Bacterial abundance (10^8^ × 16S rDNA gene copies g^−1^ soil)	7.03 ± 2.19	7.98 ± 0.96	7.01 ± 1.50	4.31 ± 1.42	7.26 ± 0.68	5.18 ± 1.01	4.50 ± 0.69	5.84 ± 0.96	7.21 ± 1.29	↑ S*

^A^The samples from July 19 were lost and could therefore not be analyzed.

**Figure 3:**
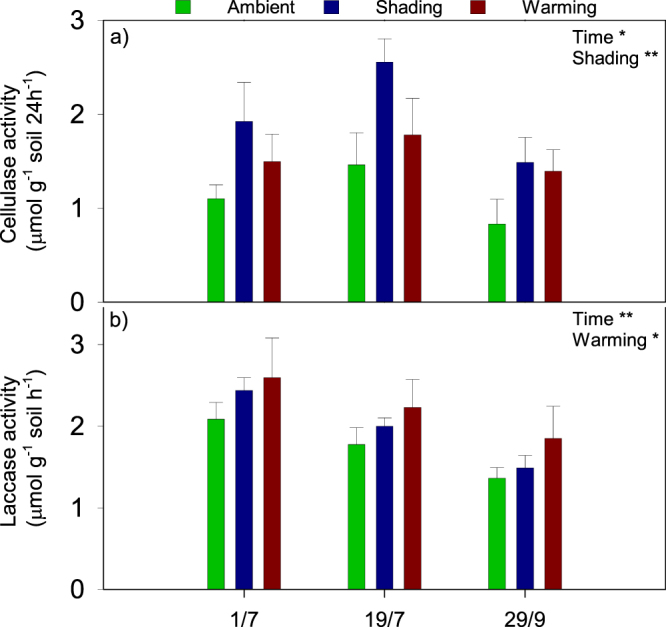
Soil enzyme activity of cellulase (**a**) and laccase (**b**) on July 1, July 19, and September 29 2013. Data are mean values obtained from six replicates ± s.e.m. The treatments were ambient, shading and warming. The statistical significant effects of the treatments and sampling day are indicated: *p < 0.05; **p < 0.01.

Fungal abundance was affected by season and peaked on September 29 (p < 0.05), yet remained unaffected by the treatments. In contrast, bacterial abundance was stable throughout the season and increased in the shading plots (p < 0.05; Table 3) concomitant to a higher cellulase activity in these plots (p < 0.01; Fig. 3a). Warming enhanced microbial activity as estimated by soil respiration (p < 0.01; Fig. 2b) and induced an increased laccase activity (p < 0.05; Fig. 3b).

### Soil fungal community responses to experimental warming and shading

**Figure 4:**
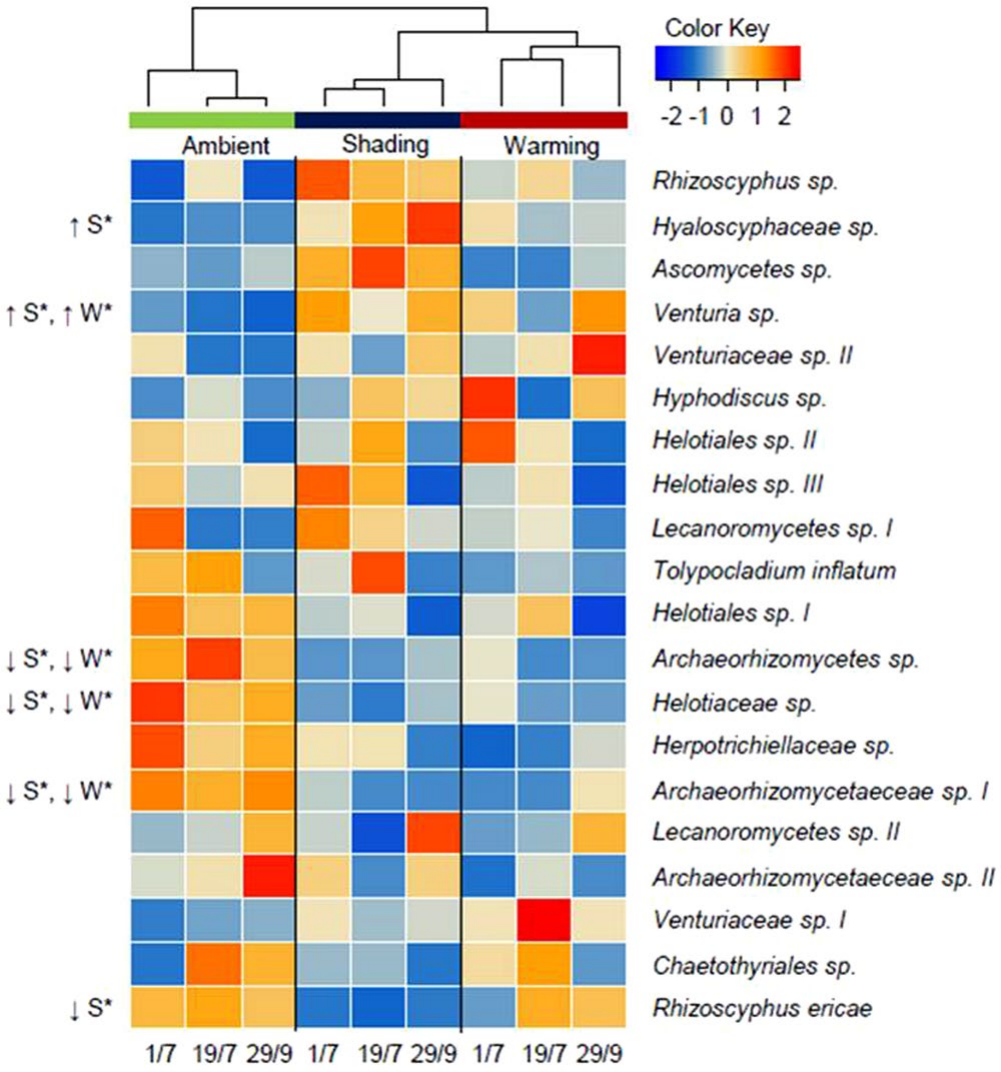
Heat map of the 20 most abundant OTUs in the soil fungal community on July 1, July 19, and September 29, 2013, after six years of shading and warming manipulations. The treatments were ambient, shading and warming, and statistical significant effects of the treatments on fungal OTUs are indicated: *p < 0.05. Arrows indicate the direction of the change.

In total, the fungal sequences were grouped into 1,400 OTUs. The community was dominated by Ascomycetes (91.6%), with a minor fraction constituted by Basidiomycetes (6.7%) and Zygomycetes (0.8%). The 20 most abundant OTUs represented 51.7% of the data set and mainly consisted of species from the orders Helotiales (*Rhizoscyphus sp*., *Hyaloschyphacea sp*., *Hyphodiscus sp*.), Pleosporales (*Venturia sp*.), and Hypocreales (*Tolypocladium inflatum*) as well as Lecaneromycetes and Archaeorhizomycetes (Fig. 4). There was no effect of sampling day on the overall structure of the soil fungal community, but six years of warming and shading altered the fungal community composition (p < 0.01, Supplementary Fig. S5). Based on the heatmap it is evident that the relative abundance of the groups *Archaeorhizomycetes sp*. and *Archaeorhizomycetaeceae sp*. *I* decreased due to shading (p < 0.05) and warming (p < 0.05; Fig. 4). The same was observed for *Helotiaceae sp*. (p < 0.01), and also the relative abundance of *Rhizoscyphus ericae* was decreased in shaded plots (p < 0.05; Fig. 4). In contrast, the relative abundance of *Venturia sp*. was higher in shading (p < 0.01) and warming (p < 0.05) plots compared to ambient plots, and likewise *Hyaloscyphaceae sp*. increased due to shading (p < 0.01; Fig. 4).

**Figure 5:**
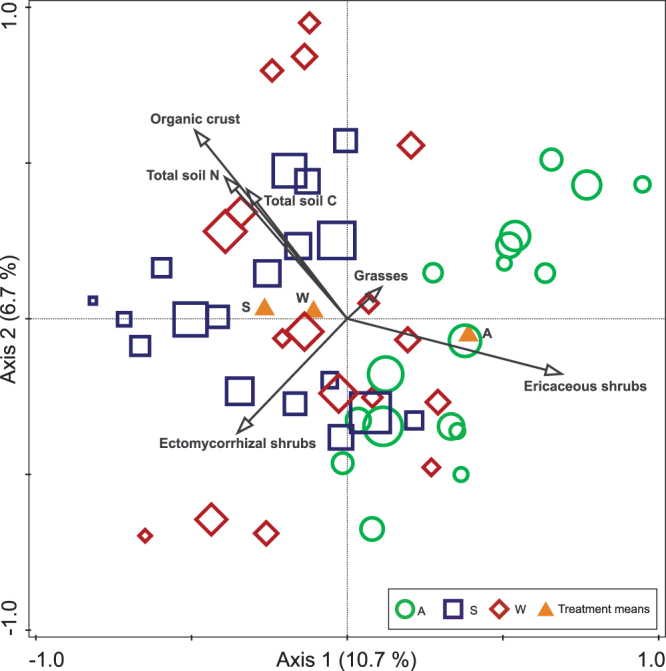
Partial redundancy analysis (RDA) of the soil fungal community on July 1, July 19, and September 29 2013 after six years of shading and warming manipulations in Kobbefjord, Greenland. The treatments were; *A* ambient, *S* shading, and *W* warming. The samples are plotted in relative size according to the number of OTUs (from 361 to 622 OTUs), and sampling date and block were covariates. Environmental variables with a significant level of p < 0.05 were included in the model (based on 1000 Monte Carlo permutations).

To identify the drivers of the fungal community structure, a redundancy analysis (RDA) with block and sampling day as covariate was done, and this indicated a significant effect of the manipulations (p < 0.01, Fig. 5). Overall, 37.9% of the variation in the fungal community could be explained in the RDA with the first two axes explaining 10.7% and 6.7%, respectively (Fig. 5). Besides an effect of the treatment, the forward selection of explanatory variables showed that cover of ericaceous shrubs (p < 0.01), organic crust (p < 0.01), total soil N (p < 0.01), coverage of grasses (p < 0.01), total soil C (p < 0.01), and ectomycorrhizal shrub cover (p < 0.01) were the factors most strongly related to fungal community composition (Supplementary Table S3).

## Discussion

Our initial hypothesis predicted a positive effect of warming on C uptake by plant photosynthetic activity^44^, but warming decreased GPP. During the entire experimental period, the net CO_2_ uptake was smaller in warming plots compared to ambient plots, which was likely caused by a reduced cover of the dominant plant species *Empetrum hermaphroditum*. Although temperature^45^ and nutrient availability^46^ are often considered the main limitations for plant growth in Arctic ecosystems, our data suggest that the warming treatment lowered soil water availability to a point where it limited plant photosynthetic activity. Because of a shallow soil layer at the experimental site, the plants have limited possibilities for water uptake in dry periods and thus, the reduced soil moisture caused by the warming treatment becomes an important determining factor for plant production. This is in agreement with previous suggestions that the role of soil moisture is underestimated when assessing the impact of climate change on Arctic ecosystems^47^.

As expected, the warming treatment increased ecosystem respiration (ER)^48^. Together with the reduced photosynthetic activity, this resulted in a dramatically reduced C sequestration. The higher ER was most likely an effect of a higher microbial activity, which was shown by enhanced soil respiration as well as higher enzymatic activity of laccase in the warmed soil. Soil fungi play a major role in tundra ecosystems as decomposers of organic matter^49^. The warming treatment changed the soil fungal community structure towards a lower relative abundance of root-associated fungi and an increase in saprotrophic fungi like *Venturia sp*.^50,51^. This change towards saprotrophic fungi may partly explain the elevated soil respiration rates despite the unaltered total abundance of fungi in the warmed soil compared to the ambient soil. Warming-induced alterations of the plant community composition were likely to be the main drivers of the observed changes to the fungal community structure. In accordance, Arctic soil fungi are mostly controlled by biological and environmental drivers such as vegetation, pH and soil moisture^52,53^, and soil microbial communties are known to be affected by plant community composition^54^. Although seasonal changes of soil microbial community compositions have been reported between summer and winter^55,56^, the fungal community composition was not affected by season in this experiment, which could be due to the fact that the most dramatic changes have been observed during spring thaw^56^, which was not part of this study.

Despite the increased C loss without a concurrent increase in C uptake, we observed no changes in the overall soil C pool. This may be due to a recent large input of organic material from the dead *E*. *hermaphroditum* plant biomass caused by the outbreak of *Eurois occulta*. Furthermore, this biomass may contain a high amount of protein-phenolic complexes that hinder decomposition as generally seen for ericaceous shrubs^57,58^, which may explain the increased laccase activity in the warming plots after six years, since laccases can decompose complex phenolic compounds^59^. However, warming did increase the soil C to N ratio after six years of warming suggesting an increased N mineralization^10^, which was supported by the higher concentrations of both DON and NO_3_^−^ in the soil. The elevated concentrations of DON and NO_3_^−^ along with elevated concentration of PO_4_^3−^ indicate that plant activity in the warmed plots was not limited by the concentration of plant available N and P.

Although shading effects on plant biomass in Arctic tundra ecosystems are previously reported to be limited^24^, we found a decrease in aboveground plant biomass and NDVI after six years of shading treatment. The shading induced decrease of *Empetrum hermaphroditum* abundance likely explains the lowered GPP. In addition, the combination of reduced ER and unaltered soil respiration in the shaded plots indicates that shading reduced aboveground plant activity and hence affected directly the C uptake capacity of the plant community.

The unaltered soil respiration rates as well as enhanced cellulase activity, higher abundance of bacteria and increase in relative abundance of *Venturia sp*. after six years of treatment contrast our initial hypothesis that shading lowers microbial activity due to lower soil temperature and reduced organic C input from litter and root exudates. The result is, however, in accordance with results from a Swedish subarctic heath^18^ and indicates that the main effect of the shading treatment at our experimental site is a lowered C uptake induced by the reduced incoming solar radiation and to a lesser extent by the lowered soil temperature.

Although the warming and shading treatments created different environmental conditions, both treatments changed the fungal community structure in a similar direction. In both climate manipulations, the relative abundance of the saprotrophic *Venturia sp*. increased, while the root-associated *Archaeorhizomycetes* groups^60^ decreased. However, the decrease in the relative abundance of root-associated fungi was more pronounced in shaded soils where e.g. the ericoid mycorrhizal species *Rhizoscyphus ericae* ^61^ decreased. Although root colonization of ericoid mycorrhizal fungi can decrease due to shading^26^, the response in the shaded plots is likely linked to the reduced cover of ericaceous shrubs. However, we are not able to establish if a decrease in *E*. *hermaphroditum* led to a decrease in *R*. *ericae*, or vice versa. The decrease in the relative abundance of mycorrhizal partners in the shaded plots may be expected to impact plant performance. On the other hand, the increased concentrations of plant available N and P in these plots may alleviate the need for a mycorrhizal partner.

The larvae outbreak by *Eurois occulta* in the fifth treatment season, 2011, lowered GPP and NEE in all plots. The combination of larvae outbreak and climate treatments significantly decreased the cover of *E*. *hermaphroditum*, while a non-significant expansion of *Salix glauca*, mosses and organic crust was observed. The increase in *S*. *glauca* cover was mainly caused by an increase in the size of individual plants and less by establishment of new plants.

Despite low numbers of moths in 2012, flower production was very low in 2012 indicating that the plants responded to the outbreak by allocating resources to growth and not to reproduction. The increased ER in the years following the outbreak (2012–2014) likely reflects an increased allocation of plant resources into growth including increased below-ground biomass. In fact, GPP was about twice as high in 2012–2014 compared to 2008–2010, which may have been facilitated by nutrients released from larval feces and carcasses^62^. A strong plant recovery following a larval outbreak was also observed in Kangerlussuaq, West Greenland^63^, indicating that tundra ecosystems are well adapted and resilient to insect outbreaks. However, the response of GPP was stronger under ambient conditions (i.e. greater difference between post- and pre-outbreak GPP) compared to the two treatments. This indicates a fast recovery of plant C uptake in ambient plots and a lowered resilience under future climate, possibly because water limitation in warming plots and light limitation in shading plots prevented a full recovery of the already stressed plant communities. In accordance, disturbed, early-succession ecosystems are considered to be more sensitive to climate change compared to mature ecosystems^27^.

Little is known about the biotic and abiotic parameters triggering an *E*. *occulta* outbreak. However, warmer climate is expected to increase the prevalence and intensity of insect outbreaks in high latitude ecosystems^64,65^. Thus, a northward expansion of moth outbreaks in northern Scandinavia is likely related to warmer winters as eggs experience enhanced winter survival^30^. Indeed, the two winters preceding the outbreak (2009/2010 and 2010/2011) were 4.0 and 2.2 °C warmer than the mean for 2008–2014.

Whether net ecosystem C storage increases or decreases in Arctic ecosystems depends on the balance between C uptake by plants and C loss from the soil organic matter pool. The predicted shrub expansion in tundra ecosystems due to warming^4^ might result in more C being bound in the aboveground woody plant biomass^66^. However, while warming slightly increased coverage of *Salix glauca* (p = 0.0984) in this study, in line with previous studies from the Arctic^4,10^, the dominant shrub *Empetrum hermaphroditum* decreased at a greater extent. The high concentration of protein-phenol complexes in *E*. *hermaphroditum* necromass may inhibit the invasion of e.g. *Salix* seedlings^57^. Thus, natural disturbances like moth outbreaks in combination with difficulties for seedling establishment following *E*. *hermaphroditum* die-back may slow down the greening of the Arctic.

The marked decrease of *E*. *hermaphroditum* after six years of warming and shading strongly affected NEE and consistently lowered C uptake in the dwarf-shrub heath. Although the fungal community composition was strongly affected by the plant community, the overall microbial activity did not follow the aboveground responses, thereby contributing to the dramatically reduced C accumulation in the dwarf-shrub heath during the growing season under a simulated future climate with elevated temperature or cloud cover. Furthermore, the reduced recovery of GPP after the larvae outbreak in the climate manipulation plots indicated enhanced ecosystem sensitivity to disturbances of Arctic ecosystems subjected to climate change. This may enhance the decoupling between above and belowground processes and further increase the sensitivity of the Arctic dwarf-shrub heath to C loss in a future changing climate where the frequency of insect outbreaks may increase.

## Methods

### Study area

The study site is an Arctic dwarf-shrub heath (vegetation height approx. 20–30 cm) dominated by the ericaceous shrub *Empetrum hermaphroditum* L. and the deciduous shrub *Salix glauca* L. with a scattered distribution of mosses, lichens, bare soil and bed rock (Table 3). The soil layer is shallow with a varying depth of 3–8 cm. The site is located in Kobbefjord approximately 20 km East of Nuuk, Greenland (64°07′N, 51°21′W), where the annual precipitation is 752 mm and the mean temperature is −1.4 °C^43,67^. In most years, the growing season is from late May to late September with snow melt occurring at day of year 148 as average for the study period. July is the warmest month with an average day temperature of 10.6 °C^43^. The annual average precipitation at the site from 2007–2014 was 859 mm (Supplementary Table S1). In the soil sampling year, 2013, snow melted in late May and the growing season began shortly thereafter and ended in late September.

### Experimental setup

The experiment was setup in 2007 as a part of the long-term Greenland Ecosystem Monitoring (GEM) program^68^ with an untreated plot (ambient, A), an open-top International Tundra Experiment (ITEX) chamber plot that increases the soil temperature by 1-2 °C (warming, W)^4,69,70^, and a shading plot (shading, S) where hessian tents remove 60% of the incoming light^68,71,72^. All plot types (1.5 × 1.5 m) are replicated in six blocks. The hessian tents over the shaded plots not only exclude incoming light but also decreased soil temperature from noon until late night (Supplementary Fig. S2c). Each year, open-top chambers and hessian tents were installed at the beginning of the growing season and taken down before first snowfall in autumn.

### Quantification of the noctuid moth *Eurois occulta*

The relative number of *E*. *occulta* larvae and other arthropods were obtained during the plant growing season by weekly sampling of eight pitfall traps at four locations within a few hundred meters of the experimental site. The traps contained ca. 200 mL H_2_O with one teaspoon of NaCl and two drops of Änglemark dishwashing detergent (Coop, Albertslund, Denmark).

### CO_2_ flux measurements

Ecosystem CO_2_ fluxes were measured weekly during the main growing season from 2008 to 2014. For exact sampling periods, see Supplementary Table S2. In 2013, the measurements took place from June 25 to September 28 by placing a plexiglas chamber (height: 0.34 m) onto a fixed metal frame (0.33 × 0.33 m) in each plot. The chamber was equipped with a HTR-2 probe logging photosynthetic active radiation and temperature during the gas flux measurements and a fan for air circulation. The probe was connected to an EGM4 (Environmental Gas Monitor, PP Systems, Amesbury, Massachusetts, USA) and a computer logging the development in CO_2_ concentration in the chamber every 10 sec (13 data-points per plot). Net ecosystem exchange (NEE) was determined under ambient light conditions. Then the flux chamber was aerated and covered with black plastic in order to determine ecosystem respiration (ER). Gross primary production (GPP) was estimated as the sum of NEE and ER.

In 2013, soil respiration was measured by means of PVC tubes (12 cm diameter; 6 cm high) placed 2-3 cm into the soil where there was no vegetation cover. The tubes were installed a week before the first measurements and soil respiration was measured as CO_2_ accumulation for 1 min using a LI-6400XT portable system together with a 6400-09 soil CO_2_ flux chamber (LICOR, Biosciences, Lincoln, Nebraska, USA). The soil respiration was hereafter measured concurrent with the chamber measurements.

### Vegetation analyses

The Normalized Difference Vegetation Index (NDVI) was measured in each plot concurrent with the CO_2_ flux measurements using a SpectroSense 2 (Skye Instruments, Llandrindod Wells, UK). In addition, the plant community composition was estimated in mid-July 2013 by pinpoint analysis in each plot using a 1 m × 1 m frame with 101 fixed points, where the number of hits on a new plant species was noted, as was hits on moss, lichen, dead crust and bare rock. The abundance of all plant species, mosses, lichens, organic crust, and bare rock was noted. Furthermore, total aboveground plant biomass was estimated for each plot based on linear regressions for each plant species between the results of a 0.35 m × 0.35 m pin-point analysis with 25 fixed points and the dry harvest weights. The latter was determined from additional plots at the experimental site, in which plant biomass was harvested and the dry weights determined.

### Soil sampling and characterization

To estimate the impact of the experimental setup, soil moisture at 2-3 cm depth was measured using a ThetaProbe Soil Moisture Sensor (Delta-T Devices, Cambridge, UK) throughout the season, and the soil temperature logged every hour 3–5 cm below the surface of each plot (n = 6) using GeoPrecision M-Log 5 W wireless temperature loggers (CiK solutions, Karlsruhe, Germany).

Soil samples (2 cm in diameter) were collected from all plots on July 1^st^, July 19^th^ and September 29^th^ 2013 by pooling three soil cores of the entire humus horizon from each plot. The soil samples were homogenized gently by hand in a metal frame, which was carefully cleaned in-between samples and 20-g soil samples were kept in sterile plastic bags at −20 °C until DNA extraction and enzyme assays were performed at laboratories in Copenhagen. Water content was determined by drying 5 g of fresh soil at 70 °C for 48 hours, whereafter soil organic matter (SOM) was determined as ignition loss when burning the dried soil at 550 °C for 6 hours. The pH was measured in a suspended solution of 5 g fresh soil in 20 mL double-distilled water (ddH_2_O).

For analysis of water extractable nutrients, 5 g fresh soil was extracted in 25 mL ddH_2_O for 1 hour to recover inorganic nitrogen (NH_4_^+^-N and NO_3_^−^-N), inorganic phosphorus (PO_4_^3−^P), dissolved organic carbon (DOC) and dissolved organic N (DON)^73^. All extracts were filtered through Whatman GF-D filters and frozen at −18 °C until further chemical analyses.

DOC was analyzed with a Shimadzu TOC-L CSH/CSN total organic C analyzer (Shimadzu, Kyoto, Japan). Inorganic N was analyzed as follows: NH_4_^+^-N using the indophenol blue method and NO_3_^−^-N colorimetrically using the cadmium reduction method, both with a FIAstar 5000 flow injection analyser (FOSS Tecator, Höganäs, Sweden). DON was analyzed with FIAstar 5000 after digesting the extracts in H_2_SO_4_ with Se as a catalyst. PO_4_^3−^ was analysed photospectrometrically by the molybdenum blue method^74^.

In addition, total soil C, N, and P were analyzed in subsamples of freeze-dried soil using a LECO TrueSpecTM CN analyzer (LECO, St. Joesph, Michigan, USA).

### Enzyme assays

Extracts for enzyme assays were prepared by shaking (150 rpm) 5 g of soil with 45 mL of sterile 0.9% NaCl for 1 h, where after 10 mL of the soil slurry was transferred to 15-mL tubes and horizontally shaken (300 rpm) with glass beads (2 mm) for 15 min. The extracts were used for assays directly. For all assays three negative controls were prepared using 5 g of autoclaved soil (randomly picked from the samples). All incubations were made in duplicates on a Gen5 2.0 Microplate Reader (BioTek Instruments, Winooski, Vermont, USA).

For the cellulase assay 200 µL soil slurry were transferred to a 96-well microtitter-plate and mixed with 200 µL substrate solution of carboxymethyl cellulose dyed with remazol brilliant blue (4 M, pH 5.0) and incubated with shaking (350 rpm) for 24 h at 40 °C. The reaction was stopped by adding 1 mL of precipitation solution (20% sodium acetate trihydrate and 3% zinc acetate in 100 mL milliQ water; pH adjusted to 5.0 by HCL), incubated for 10 min at room temperature, centrifuged, the supernatant (200 µL) transferred to a new microtitter-plate, and the absorbance measured at 590 nm^75^.

For laccase, 20 µL of soil slurry were transferred to a 96-well microtitter-plate and mixed with 50 µL of ABTS (2,2′-azino-bis(3-ethylbenzthiazoline-6-sulfonic acid)) (50 mM) and 130 µL sodium-acetate buffer (100 mM, pH 5.0). The plate was incubated with shaking (100 rpm) for 1 h at room temperature and then centrifuged at 3500 rpm for 4 min. The supernatant (200 µL) was transferred to a new plate and absorbance measured at 420 nm^75^.

### DNA extraction and quantitative PCR

DNA was extracted from 0.25 g freeze-dried soil using FastDNA Spin Kit for Soil, according to manufacturer’s standard protocol (MP Biomedicals, Solon, Ohio, USA). The DNA was kept at −18 °C and used for both quantitative PCR (qPCR) and PCR amplicon sequencing.

Fungal and bacterial abundance in the soil was estimated by qPCR using 20 µL reactions on Mx3000 P (Agilent Technologies, Santa Clara, California, USA) targeting the ITS2 region for fungi using ITS4^76^ and ITS7^77^ primers, and targeting bacterial 16S rDNA by EUB338F and EUB518R primers^78^. The qPCR reactions were run in technical duplicates and contained 10 µL of SYBR® Green QPCR Master Mix, 2 µL of each primer (770 nM final concentration), 1 µL of DNA template, and 5 µL of ultraclean water (Sigma-Aldridge, St. Louis, Missouri, USA). The qPCR program for 16S rDNA amplification was: 95 °C for 3 min followed by 40 cycles of 95 °C for 10 s, 60 °C for 20 s, and a final dissociation curve. The standard curve for bacteria was made from *E*. *coli* sø3339. The qPCR program used for amplification of the ITS region was: 94 °C for 10 min followed by 40 cycles of 94°C for 30 s, 56 °C for 30 s and 72 °C for 1 min, and a final dissociation curve. The standard curve for fungi was made from *Aureobasidium pullulans*. After the sequencing results of the ITS region were obtained, the qPCR results were adjusted for amplified plant DNA.

### Sequencing of soil fungal community

The primers ITS4 and ITS7 were used to amplify the ITS2 region for sequencing. PCR amplifications were done in two steps. The first step using illustra puReTaq Ready-To-Go PCR Beads (GE Healthcare, Little Chalfont, UK) only added primers and template DNA (1 µL). The PCR-I conditions were 94 °C for 2 min; 35 cycles of 94 °C for 30 s, 56 °C for 30 s, 72 °C for 30 s, followed by 72 °C for 5 min. The second step was done using 2 µL of the 10x diluted PCR-I product in a reaction mixture including 0.15 µL DNA polymerase (AccuPrime™ Taq DNA Polymerase High Fidelity, Invitrogen, Thermo Fisher Scientific, Waltman, Massachusetts, USA), 2 µL 10x AccuPrime™ PCR Buffer II, 1 µL (770 nM) of the each primer (custom tagged forward and reverse primer), and 13.85 µL ddH_2_O to a 20 µL reaction volume. The conditions for PCR-II were 94 °C for 2 min; 14 cycles of 94 °C for 30 s, 56 °C for 30 s, 72 °C for 30 s, followed by 72 °C for 5 min. Amplicons were then purified from a 1% agarose gel using Montage Gel Extraction Kit (Millipore, Billerica, Massachusetts, USA). Amplicon concentrations were quantified for all samples using Qubit dsDNA HS Assay and measuring the fluorescence on Qubit fluorometer (Invitrogen). Samples were pooled in equimolar amounts and paired-end sequenced on a MiSeq (Illumina, San Diego, California, USA).

### Processing sequence data

Sequences were processed using Qiime 1.7^79^. Sequences were trimmed of primer sequences and barcodes and assembled using a custom script. Sequences that could not be assembled were removed as well as chimeras and sequences with a quality score less than 20. The remaining sequences (6.5 × 10^6^ reads with an average read length of 435 bp) were clustered into operational taxonomic units (OTUs), using Usearch 5.2.236^80^ at 97% similarity level in Qiime. OTU-clusters containing less than 100 reads were removed. The remaining OTUs were classified using RDP classifier^81^ against the UNITE database^82^.

The result of this classification contained two major groups ‘Fungi sp.’ and ‘Fungi, Ascomycota sp.’ containing 575 OTUs with > 100 reads and accounting for 23.5% of the data. These were BLASTed through the NCBI NT database, in an attempt to obtain a higher taxonomic resolution. Alignment parameters were modified from default: Word size = 11, reward = 2, gapopen = 4, gapexted = 2. Hits with e-value < 10^−5^ were discarded. Taxonomy was voted upon with the BROCC^83^ 1.1.0 pipeline with the default ‘amplicon’-mode parameters. This resulted in re-classification of 113 OTUs, accounting for 11% of the dataset. Another 113 OTUs (0.25%) were classified outside the fungal kingdom and were removed from the dataset. 349 OTUs (12%) did not obtain better classification. All annotations in the classification of ‘other’, ‘unknown’, ‘unidentified’, ‘uncultured’ and ‘Incertae sedis‘ were designated as ‘unknowns’ even though a categorical difference in these terms do exist.

After all removals, the dataset consisted of 4.1 × 10^6^ reads, 1,400 OTUs and 151 taxonomic groups. Sample 4 W and 6 C from July 1^st^ contained low amounts of assembled reads and were removed from the dataset. The remaining samples were rarefied to 8,680 reads per sample before the between-sample community analysis. The sequences are available from GenBank with accession numbers KR265904 - KR267303.

### Statistical analyses

Analyses of variance (ANOVA) were done using SAS 9.3 (SAS Institute, Cary, North Carolina, USA). The effects of nutrient concentrations, enzyme activity, plant biomass, vegetation abundance, and fungal and bacterial abundance were analyzed by linear mixed model with block as random factor. A similar model was used for analyzing the effects of sampling day. Time series of soil moisture, ecosystem respiration, soil respiration, soil temperature, net ecosystem exchange and NDVI were analyzed by repeated measurement ANOVA with block as random factor. Dunnett’s test was used to identify the difference between ambient (control conditions) and either shading or warming treatments. All significant (p < 0.05) results are reported in figures and tables.

A between-groups analyses of the fungal community composition in the different treatments was performed in R^83^ using the package ‘ade4’^84,85^ based on a Euclidean distance matrix of the rarefied OTU table, with 10^6^ Monte-Carlo permutations. In addition, a heatmap of the 20 most abundant OTUs was produced in R using the packages ‘vegan’^86^ and ‘rioja’^87^. Furthermore, the treatment effects on theses 20 clusters were analyzed by STAMP v. 2.0.9^88^.

A redundancy analysis (RDA) was done in CANOCO 5 (Microcomputer Power, Ithaca, New York, USA) with sampling day and blocks as covariates, and explanatory factors were accepted by forward selection with a significant level of p < 0.05 based on 1000 Monte Carlo permutations followed by Bonferroni correction of the p-values. All analyzed environmental factors were included in the forward selection except for CO_2_ flux measurements, fungal abundance and enzyme activity. Response variables (rarefied OTU abundance) were arcsine-transformed and centered as recommended by Ramette^89^. Explanatory variables (environmental data) were centered and standardized by z-score calculations as default in CANOCO 5^90^.

The ITS2 sequences generated during the current study are available in GenBank with accession numbers KR265904 - KR267303, while other data generated during the current study are available from the corresponding author on reasonable request.

## Electronic supplementary material

Supplementary Information
