# Supplementary material for: Warming, shading and a moth outbreak reduce tundra carbon sink strength dramatically by changing plant cover and soil microbial activity
Source: Sci Rep. 2017 Nov 22;7:16035. doi: 10.1038/s41598-017-16007-y (PMC5700064; doi:10.1038/s41598-017-16007-y)
Supplement: Supplementary file 1 — Supplementary Information [file 41598_2017_16007_MOESM1_ESM.pdf]

Supplementary Information

Warming, shading and a moth outbreak reduce tundra carbon sink strength dramatically by changing plant cover and soil microbial activity

List of authors:
Mathilde Borg Dahl (mathildeborg.dahl@uni-greifswald.de)
Anders Priemé* (aprieme@bio.ku.dk)
Asker Brejnrod (asker.brejnrod@bio.ku.dk)
Peter Brusvang (peter.brusvang@hotmail.com)
Magnus Lund (ml@bios.au.dk)
Josephine Nymand (jony@natur.gl)
Magnus Kramshøj (magnus.kramshoej@bio.ku.dk)
Helge Ro-Poulsen (helgerp@bio.ku.dk)
Merian Skouw Haugwitz (mehau@dtu.dk)

The following Supplementary Information is available for this article:

Supplementary Figure S1 Number of days with different soil moisture contents (%) during the growing season 2008-2014.

Supplementary Figure S2 Temperature (ºC) measurements from the experimental site in 2013.

Supplementary Figure S3 Net ecosystem exchange (NEE) in 2011 during the larvae outbreak.

Supplementary Figure S4 Correlation between aboveground plant biomass and NDVI in July 2013.

Supplementary Figure S5 Principle component analysis (PCA) of a between group analysis of the soil fungal community.

Supplementary Table S1 Total annual precipitation and precipitation during the growing season at the experimental site from 2007-2014.

Supplementary Table S2 Exact sampling period for CO_2 flux measurements.

Supplementary Table S3 Results of the RDA forward selection analysis of the soil fungal community structure.

Supplementary Figure S1 Number of days with a specific average soil moisture content measured during the growing seasons concurrent with CO_2-flux measurements (Fig. 1) from 2008 to 2014. The climate change experiment at Kobbefjord, Greenland, consisted of three different treatments; ambient, shading, and warming. The presented data include 1232 sampling days from each treatment.

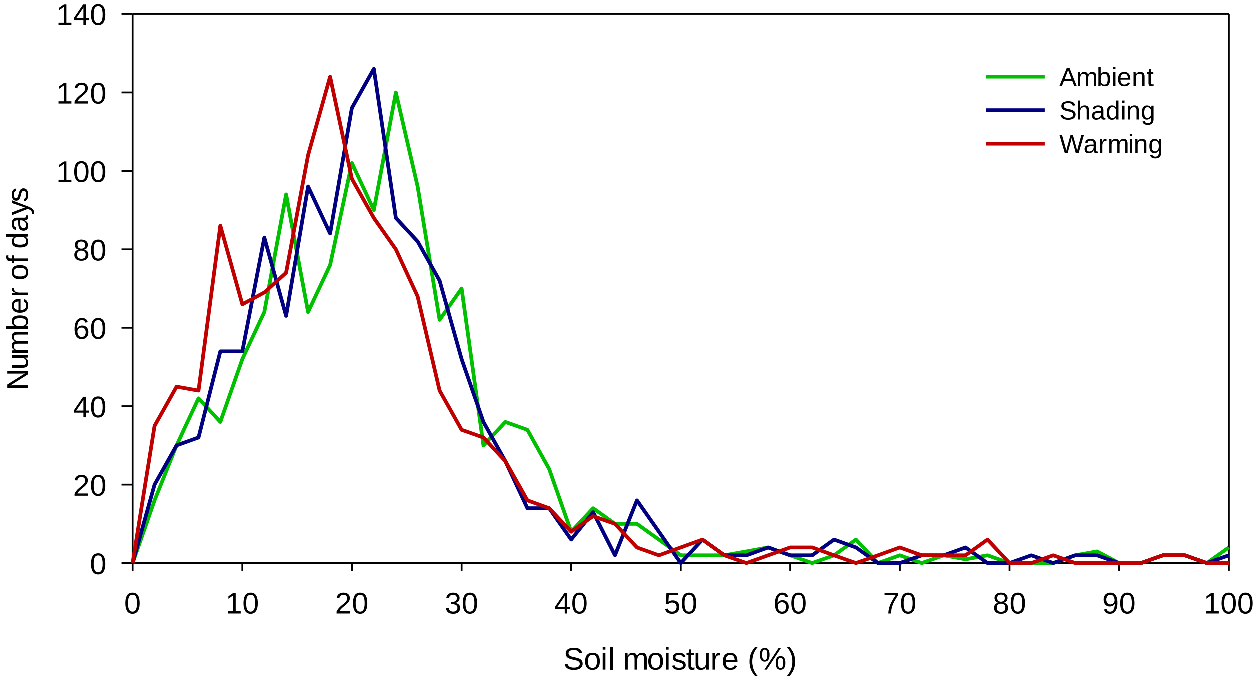

Supplementary Figure S2 Daily average of soil temperature at 2-3 cm soil depth from 2013 (a), daily average soil temperature during the sampling period June to end-September 2013 (b), and daily fluctuations in soil temperature from July 2013 (c). The climate change experiment at Kobbefjord, Greenland, consisted of three different treatments; ambient, shading, and warming. The statistical significant effects of the treatments are indicated: **$p < 0.01$.

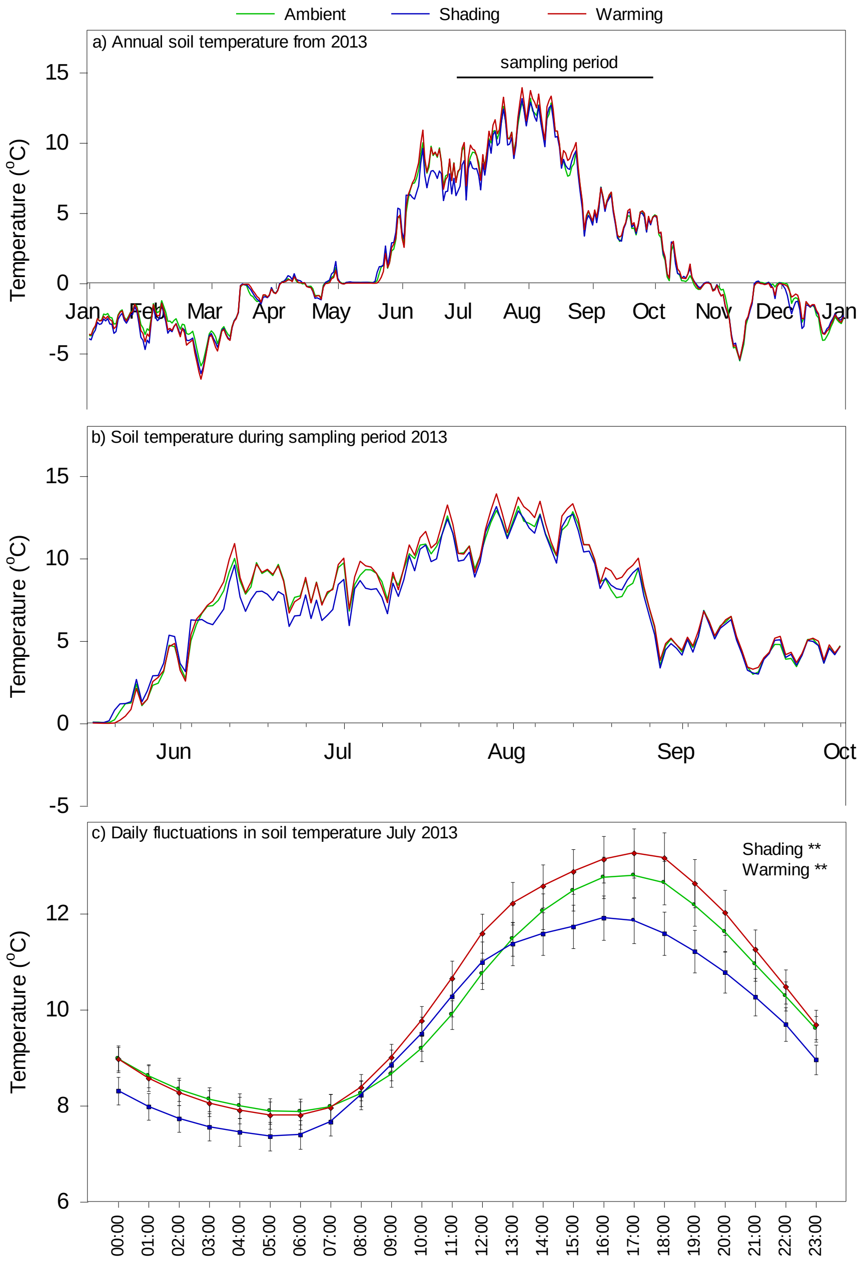

Supplementary Figure S3 Net ecosystem exchange (NEE) measured under mid-day conditions during the growing season 2011 at the experiment site in Kobbefjord, Greenland. The photosynthetic activity was low that year because of a larvae outbreak of the noctuid moth *Eurois occulta*, which resulted in a dramatic set back of the vegetation in July due to inhibition of the production of leaves, buds and flowers. The number of larvae peaked between the 28th of June to the 5th of July, when 1859 individuals were collected from eight pitfalls in an 8 x16 m area. The three different treatments were; ambient, shading, and warming. The statistical significant effects of time and treatments are indicated: **p < 0.01.

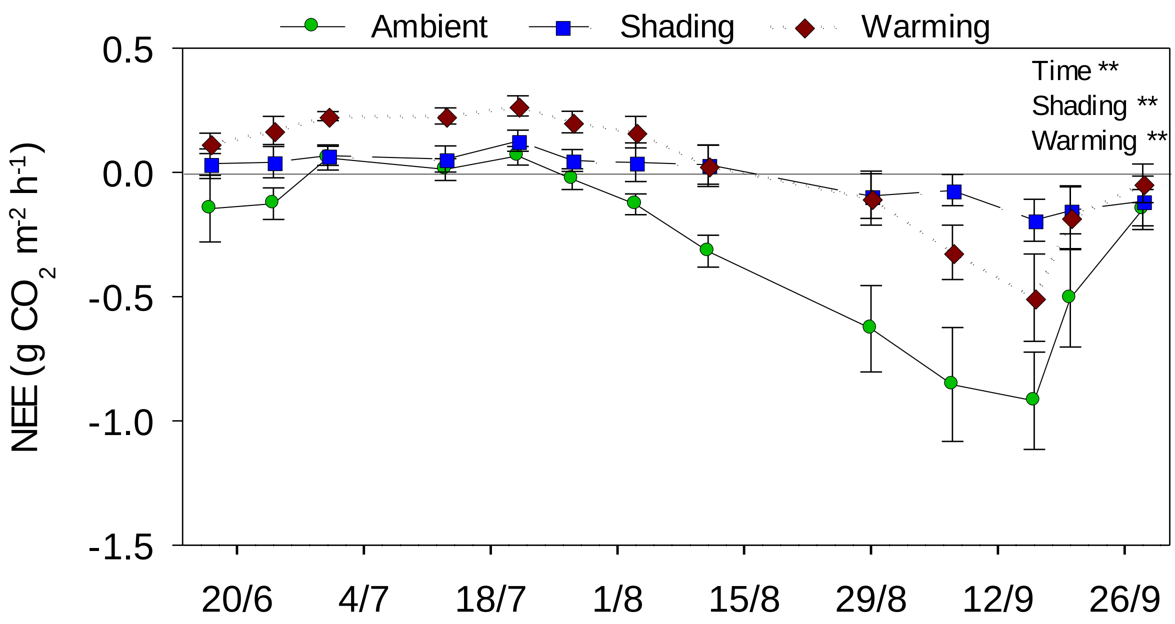

Supplementary Figure S4 Correlation between total aboveground plant biomass (g m^{-2}) and NDVI at the experimental site in July 2013. All samples are shown (n = 18). The solid line is the regression line (r^2 = 0.49) and the dotted lines indicate the 95 % confidence intervals.

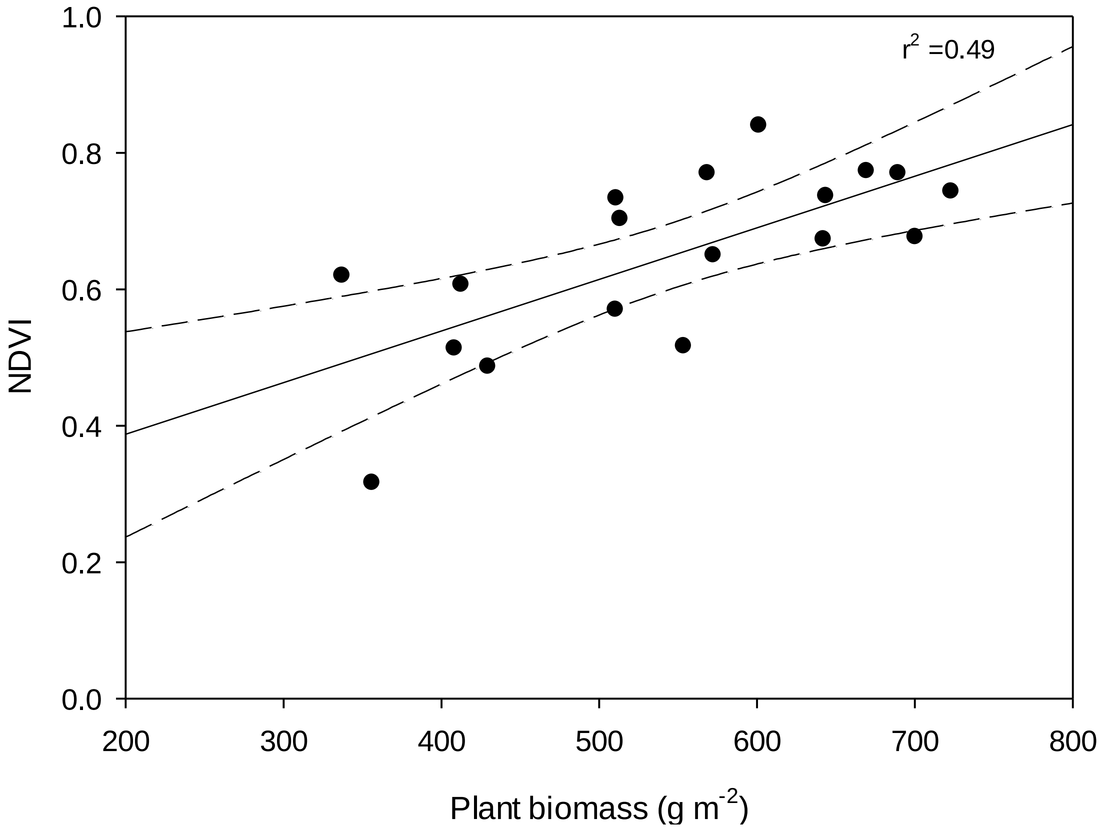

Supplementary Figure S5 Principle component analysis (PCA) of a between group analysis of the soil fungal community after six years of warming and shading treatments. The PCA is based on 100,000 Monte Carlo permutations and the ratio indicates the preserved Euclidean distance between samples after grouping (interval [0-1]). The treatments were; ambient, shading, and warming.

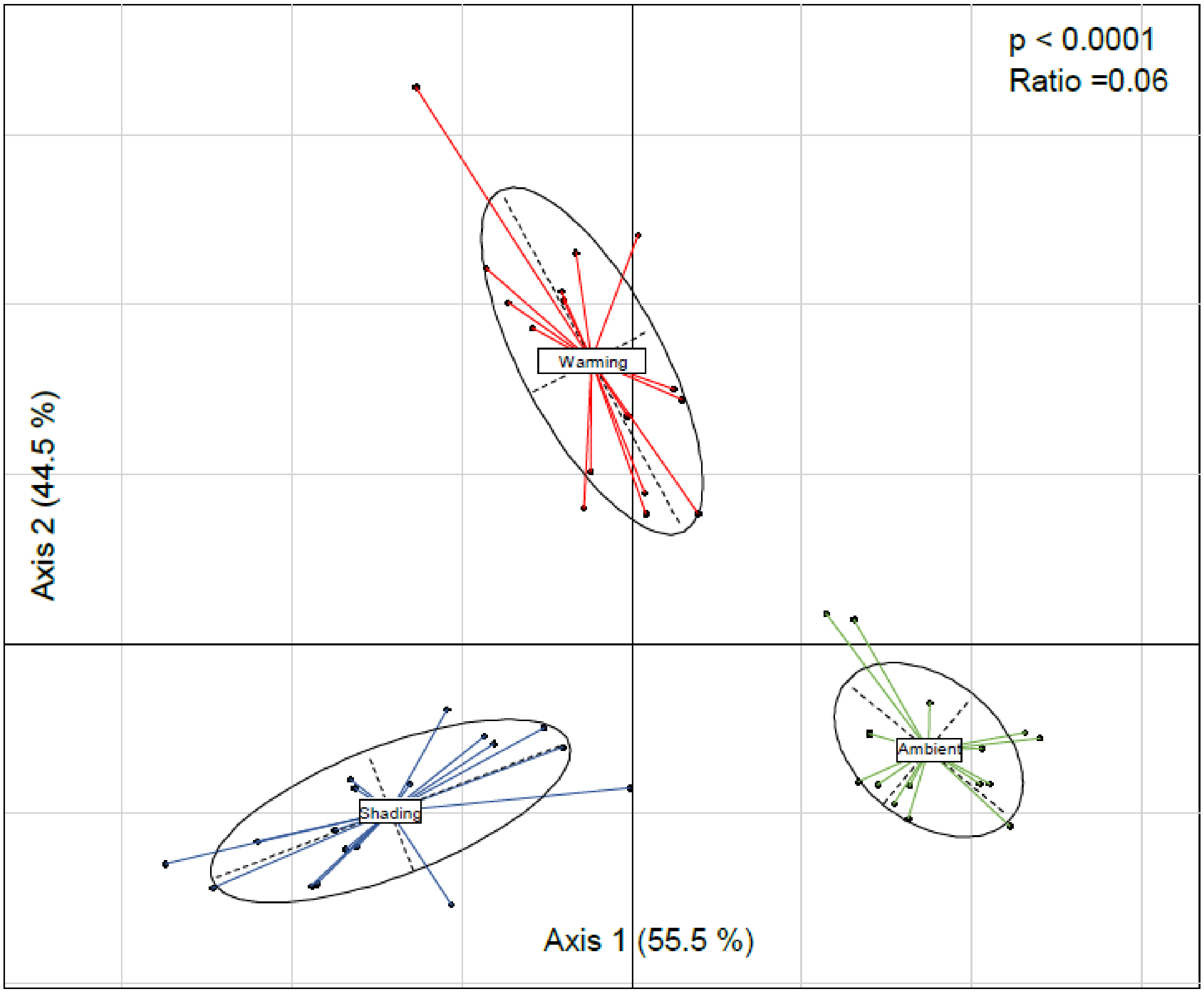

Supplementary Table S1 Total annual precipitation and precipitation during the CO2-flux measurement period from early June to end-September at the experimental site at Kobbefjord, Greenland. The data are shown for each year from 2007 to 2014 and as average for 2007-2014.

	2007	*2008*	*2009*	*2010*	*2011*	*2012*	*2013*	*2014*	*Average*
Annual precipitation (mm)	658	1064	844	932	541	1067	1054	709	859
Precipitation June-September (mm)	360	252	187	362	234	476	434	381	336

Supplementary Table S2 Exact period for the CO2 flux measurements at the experimental site at Kobbefjord, Greenland. All eighteen plots (three treatments and six replicates of each) were measured at each sampling time.

	2008	*2009*	*2010*	*2011*	*2012*	*2013*	*2014*
Start date	June 25th	June 23rd	June 23rd	June 24th	June 22nd	June 25th	June 18th
End date	Sept. 23rd	Sept. 23rd	Sept. 28th	Sept. 28th	Sept. 28th	Sept. 28th	Sept. 26th

Supplementary Table S3 Results from the RDA forward selection of significant explanatory variables, their contribution to explaining the model, and their p-values based on 1000 Monte Carlo permutations followed by Bonferroni correction. Overall, the selected explanatory variables explained the entire RDA model, which represented 37.9 % of the variation in the soil fungal community.

	Contribution (%)	*F-value*	*P (adjusted)*
Ericaceous shrubs (% coverage)	22.8	4.1	0.0023
Organic crust (% coverage)	16.1	3.0	0.0023
Total soil N (mg g^{-1} soil)	13.0	2.5	0.0018
Grasses (% coverage)	11.0	2.2	0.0013
Shading treatment	11.3	2.3	0.0011
Total soil C (mg g^{-1} soil)	9.5	2.1	0.0015
Ectomycorrhizal shrubs (% coverage)	8.5	1.8	0.0030
Warming treatment	7.9	1.7	0.0067
